# Differential gene expression analysis following olfactory learning in honeybee (*Apis mellifera* L.)

**DOI:** 10.1371/journal.pone.0262441

**Published:** 2022-02-09

**Authors:** Muhammad Fahad Raza, Muhammad Anwar, Arif Husain, Muhmmad Rizwan, Zhiguo Li, Hongyi Nie, Pavol Hlaváč, M. Ajmal Ali, Ahmed Rady, Songkun Su

**Affiliations:** 1 College of Animal Sciences (College of Bee Science), Fujian Agriculture and Forestry University, Fuzhou, China; 2 College of Life Sciences, Fujian Agriculture and Forestry University, Fuzhou, China; 3 Guangdong Technology Research Center for Marine Algal Bioengineering, Guangdong Key Laboratory of Plant Epigenetics, College of Life Sciences and Oceanography, Shenzhen University, Shenzhen, China; 4 Department of Soil and Environmental Sciences, Faculty of Agricultural Sciences, Ghazi University Dera Ghazi Khan, Dera Ghazi Khan, Pakistan; 5 Department of Integrated Forest and Landscape Protection, Faculty of Forestry, Technical University in Zvolen, Zvolen, Slovakia; 6 Department of Botany and Microbiology, College of Science, King Saud University, Riyadh, Saudi Arabia; 7 Department of Zoology, College of Science, King Saud University, Riyadh, Saudi Arabia; King Khalid University, SAUDI ARABIA

## Abstract

Insects change their stimulus-response through the perception of associating these stimuli with important survival events such as rewards, threats, and mates. Insects develop strong associations and relate them to their experiences through several behavioral procedures. Among the insects, Apis species, *Apis mellifera ligustica* are known for their outstanding ability to learn with tremendous economic importance. *Apis mellifera ligustica* has a strong cognitive ability and promising model species for investigating the neurobiological basis of remarkable olfactory learning abilities. Here we evaluated the olfactory learning ability of *A*. *mellifera* by using the proboscis extension reflex (PER) protocol. The brains of the learner and failed-learner bees were examined for comparative transcriptome analysis by RNA-Seq to explain the difference in the learning capacity. In this study, we used an appetitive olfactory learning paradigm in the same age of *A*. *mellifera* bees to examine the differential gene expression in the brain of the learner and failed-learner. Bees that respond in 2^nd^ and 3^rd^ trials or only responded to 3^rd^ trials were defined as learned bees, failed-learner individuals were those bees that did not respond in all learning trials The results indicate that the learning ability of learner bees was significantly higher than failed-learner bees for 12 days. We obtained approximately 46.7 and 46.4 million clean reads from the learner bees failed-learner bees, respectively. Gene expression profile between learners’ bees and failed-learners bees identified 74 differentially expressed genes, 57 genes up-regulated in the brains of learners and 17 genes were down-regulated in the brains of the bees that fail to learn. The qRT-PCR validated the differently expressed genes. Transcriptome analyses revealed that specific genes in learner and failed-learner bees either down-regulated or up-regulated play a crucial role in brain development and learning behavior. Our finding suggests that down-regulated genes of the brain involved in the integumentary system, storage proteins, brain development, sensory processing, and neurodegenerative disorder may result in reduced olfactory discrimination and olfactory sensitivity in failed-learner bees. This study aims to contribute to a better understanding of the olfactory learning behavior and gene expression information, which opens the door for understanding of the molecular mechanism of olfactory learning behavior in honeybees.

## Introduction

Insects are essential for agriculture, orchards, horticultural crops, and seed production for fiber and forage crops [1]. Insects such as bees, butterflies, wasps, and beetles are increasing the output of the world’s food crop production. Most insects learn about the ecologically related stimuli and modify the behavioral responses to these stimuli to accept the new associations in their ecosystem. Diverse methods can trigger these behavioral changes due to new stimuli [2, 3]. These plasticity types vary how the behavior and stimuli are linked with significant events, including the availability of food, danger, or a mate. It is quite essential to understand these mechanisms of plasticity for preliminary research on how it influences the nervous system’s ability to process olfactory learning [4, 5]. The evaluation of behavioral plasticity in any organism involves an experimental level of control throughout several factors, which is not accomplishable in field conditions to conduct behavioral research in natural circumstances, it is necessary to develop a standard conditioning procedure that can be performed under controlled conditions while still being useful [6].

The *A*. *mellifera ligustica* is a well-known and excellent insect for, how to establish a standard protocol for carrying out a study of behavioral plasticity under controlled conditions [6–8]. In honeybees, the proboscis extension response is a natural response in which they elongate their proboscis (elongated feeding tube) by touching the antenna with the sucrose solution. Proboscis extension response (PER) is established whenever honeybees find nectar in flowers during foraging behavior. Interestingly, the honeybee will promptly show this easy and simply assessable behavior under laboratory conditions. It enables us to investigate the mechanisms under a controlled setting that influence this environmental significance behavior [9]. In standard conditioning protocol, PER can be applied to evaluate the learning, stimulus perception, and memory under the various experimental conditions which are developed to disclose the neural and behavioral mechanisms that underlie plasticity [10]. In 1957, since the first study proboscis extension protocol was performed by Kuwabara [11], PER protocol has been extensively used to investigate the operant, associative and failed-associative mechanism that basic learning behavior of the honeybee [12]. The proboscis extension reflex (PER) experiment was carried out for the first time by Takeda in 1961, who combined an olfactory stimulus with a sucrose reward [13].

A universal distinctive feature of sensory perception is its dynamic nature. It continually accepts the ecological situation, that instigates different variations in behavioral response and odor perception and processing. The cognition behind this dynamical nature is almost omnipresent in the sensory pathways impacting the entire level of olfaction circuits, from sensory receptors to sensory centers and primary neuropils [14]. Many studies on honeybees also exhibited that conditioning trials interval also affects learning and memory formation [15]. Honeybees are trained, rewarded with sucrose solution with three consecutive conditioning trials with inter-trial interval -10 min. Where a stimulus odor takes 4 seconds and is associated with a food reward to evaluate the learning performance [16], Honeybees have excellent olfactory learning abilities. Bees learn about odor through olfactory receptor cells which are present on their antenna, changing these odor cues to chemical signals and subsequently transferring them into the mushroom body [17, 18]. The classical experimentation design for olfaction learning has been progressively improved [19]. In bees, several studies have been done on learning skills in the honeybee, the underlying molecular process is still unknown, particularly for olfaction behavior by using a stimulation device controlled by a computer. Up to now, only a few genes have been identified as being involved in the learning process of honey bees [20, 21].

For molecular studies of social behavior, *A*. *mellifera* is known as an ideal insect [22, 23]. The genome of *A*. *mellifera* provides us with valuable evidence that significantly promotes the intriguing study of future olfactory research [24]. Natural learning selection has developed the bee brain to link signals that prognosis the availability of nutrient-rich foods. Sensory perception is coordinated to develop cognitive traces of stored nutriment (food) for restoration if bees are starving so that these honeybees can recognize stimuli related to nutritious food rewards. Post-ingestive signaling is an essential system for evaluating food importance and developing sensory signals with long-term memory [25]. The most excellent and crucial function of the bee’s brain is to remember and learn about the tasks connected with sucrose solution and food [26]. In honeybees, olfactory learning, particularly odor learning is supposed to form by integrating sensory information into the mushroom bodies (prominent and outstanding part in the brain) [27]. Gene expression has been studied in the brain of bees to identify genes that have a major function in olfaction behavior. Certain genes are mainly expressed in the intrinsic neurons named Kenyon cells in the mushroom body [28].

In previous studies, the researchers examined olfactory associative learning performance by using PER conditioning protocol [7, 29]. According to our investigation, the system for comparing the gene expression profiles of the 12-days old bees with unique olfaction learning skills. The first time, we used an olfaction stimulation device controlled by a computer to evaluate the performance of olfactory learning behavior of same-age honeybees. In the article, we analyzed the patterns of genes expressed in the brain of the learner and a failed-learner group of bees to display and identify the differentially expressed genes responsible for the learning ability development of honeybees. The goal of this experiment to transcriptome of bees’ brain after olfactory conditioning trials to test the hypothesis that learner bees have high expression of genes as compared to failed-learner bees involved in olfactory learning behavior, also find the genes involved in olfactory learning behavior by adopting the DEG approach at genome wide level. We also tested the learning performance of *A*. *mellifera legustica* because honeybees have a major ecological role as pollinators in multiple ecosystems. We analyzed the transcriptome of individual bee brains of learner and failed learner bees using RNA-Seq to provide the robust analysis of learner and failed learner bees. To confirm the RNA sequencing outcomes, quantitative real-time-PCR (qRT-PCR) was performed.

## Material and methods

### Experimental location and handling of bees

*A*. *mellifera ligustica* bees were obtained from the experimental apiary of the College of Animal of sciences, Fujian Agriculture and Forestry University, China (26°05’9.60" N 119°14’3.60" E). Capped combs were collected from three different healthy colonies and kept in an incubator. The newly emerged bees were obtained every day and were transferred into ten laboratory rearing glass cages (Number of bees/cages = 48). In an incubator (30°C, RH -70%), the caged bees were placed and fed for 12 days with sucrose solution 30% and a mixture of sucrose solution 50%, and pollen. When the bees were 12 days old, they were brought to the laboratory for olfactory PER conditioning.

### Olfactory proboscis extension response conditioning

Bees were harnessed following a standard procedure [30, 31]. and kept in an incubator at a temperature of 30°C and relative humidity of 70% (±1, 70%) for one hour. Before conditioning, a drop of 30% (w/v) sucrose solution was delivered to the antennae to check for intact PER. Bees not responding with PER to this stimulation were discarded from the experiment. Population responses of bees (n = 251) were trained to discriminate between the learner and failed-learner during three conditioning trials. Twelve old days’ bees were used to discriminate the olfactory learning trials. Bees were trained using a conditioning procedure to discriminate learner and failed-learner by using 1-nonanol (Sigma Aldrich, France) paired with sucrose solution 30%. Each conditioning trial lasted 39 sec. First, the harnessed bee was positioned in front of the olfactometer, and clean air was delivered to the antennae during 15 s. An odorant was then delivered in 4 sec. Two sec after odor onset, sucrose solution was delivered for 2 sec. Thus, the interstimulus interval was 2 sec and the Conditioned stimulus and unconditioned stimulus ended at the same time. Finally, clean air was delivered in the absence of other stimulations for 20 sec to complete the 39-sec trial. The intertrial interval was 10 min.

At the end of the learning trials, individuals that respond in the 2^nd^ and 3^rd^ trials or only responded to 3^rd^ trials were defined as learned bees, failed-learner individuals were those bees that did not respond in all learning trials. After the third learning trial, samples from the learner and failed-learner groups of honeybees were stored at 80°C until brain dissection after being frozen in liquid nitrogen [32].

### Library preparation and RNA sequencing

Three repeated samples were used for learner and failed-learner bees, respectively. Total RNA contamination and degradation were examined by denaturing gel electrophoresis (1.0%). Purity and total RNA concentration were measured and checked by Qubit® RNA Assay Kit in Qubit® 2.0 Fluorometer (Life Technologies, CA, USA) and Nano Photometer® spectrophotometer (IMPLEN, CA, USA) respectively. Moreover, the integrity of RNA was validated using the RNA Nano 6000 Assay Kit of the Bioanalyzer 2100 system (Agilent Technologies, CA, USA). Concisely, the mRNA was purified from total RNA with a magnetic beads oligo. Fragmentation was performed under elevated temperature using the divalent cationed in NEB Next First-Strand Synthesis Reaction Buffer (5X). The first cDNA strand (from the fragmented mRNA) was synthesized with random hexamer primers using M-MuLV Reverse Transcriptase (RNase H-). Using DNA polymerase I and RNase H, second-strand cDNA synthesis was consequently performed. Polymerase/exonuclease activities changed the lasting overhangs into blunt ends. NEB Next Adaptor with hairpin loop structure was ligated to prepare for hybridization after adenylation of 3 "ends of DNA fragments. To select cDNA fragments of 150~200 bp, preferably in length, With the AMPure XP system (Beckman Coulter, Beverly, USA), and the library fragments were purified. Therefore, 3 μl USER Enzyme (NEB, USA) was used with selected size and adaptor-ligated cDNA at 37°C for 15 minutes succeeded by 5 minutes at 95°C before polymerase chain reaction. The PCR was carried out using Phusion High-Fidelity DNA polymerase, Universal PCR primers, and Index (X) Primer. PCR products (AMPure XP system) were lastly purified, and library quality on the Agilent Bioanalyzer 2100 system was measured.

### Illumina sequencing, assembly, and annotation

Index-coded samples were clustered using TruSeq PE Cluster Kit v3-cBot-HS (Illumina) per provided by the manufacturer requirements in a cBot Cluster Generation System. The library preparation was sequenced on the platform of Illumina Hiseq after the cluster generation. The paired-end reading ranged in length from 125 to 150 bp. Clean reads were filtered by discarding the adopter sequence, and reads were discarded when more than 10% (N > 10%) of either read was unsure nucleotides. Ultimately, remove the reads when nucleotides of low quality (basic quality below 20) constitute over 50% of the reading. The differential gene expression was carried out through the abundance of transcripts. The higher the abundance, the higher the level of gene expression. The level of gene expression was calculated in our RNA sequence assessment by counting the reads that map to genes or exons. Not only is the read count proportional to the actual level of gene expression, but it is also comparable to the length of the gene and the depth of sequencing. The FPKM was used to compare the levels of gene expression estimated from different experiments. In RNA—sequence, FPKM (Fragments Per Kilobase of transcript sequence per Millions of base pairs sequenced) is the most standard method of estimating levels of gene expression, considering the effects of both sequencing depth and gene length on fragment counting. Differential gene expression (DEGs) was performed using DESeq (R package) between learner and failed-learner libraries. Directed Acyclic Graph (DAG) is a way of showing Gene Ontology (GO) enrichment results for differentially expressed genes (DEGs). The enrichment pathway analysis identifies metabolic pathways or signal pathways significantly enriched with differentially expressed genes compared to the entire genome [33].

N is the number of all genes with a KEGG annotation, n is the number of DEGs in N, M is the number of all genes annotated to specific pathways, and m is the number of DEGs in M.

### Analysis of quantitative real-time-PCR (qRT-PCR)

To confirm the RNA sequencing outcomes, the integumentary system, storage proteins, brain development, sensory processing, and genes from enriched KEGG pathways and GO categories were analyzed through quantitative real-time PCR (qPCR). cDNA was synthesized using the RT mentioned above Reagent Kit with gDNA Eraser (Takara, China) from RNA samples gained as directed by the manufacturer. Mixed Total RNA (1 μg) with 2 μl (5 x gDNA Eraser). RNase-free water and 1μl of gDNA Eraser and a final volume of 10μl was added. To eliminate genomic DNA, the incubation of the reaction mixture for 2minutes at 42°C. The reaction blends mixed in 4 μl 5 μl PrimeScript,1 μl RT Primer,4 μl RNase Free water, 1 μl Prime Script RT enzyme, incubated for 5s at 85°C and 2 minutes at 37°C. The prepared complementary DNA (cDNA) was stored at -20°C for subsequent use. Every qPCR-10 μl reaction was made up of 5 μL of 2x SYBR Premix Ex Taq II (Tli RNase H Plus, TaKaRa), 0.8μl of every primer (10 μM) first, cDNA1 μl (1:3 dilution), and RNase-free water 3.2 μl. The Bio-Rad CFX 384 real-time system was used to perform qPCR, and every reaction was repeated three times. The reaction conditions were for the 30 sec at 95°C and 40 Cycles for 5 sec at 95°C and 30 sec at 60°C, followed by the analysis of the melting curve. Each target’s Ct values were normalized to the geometric mean of one’s Ct values [33].

### Statistics

For RNA-Seq data analysis, the resulting P-values were adjusted using the Benjamini and Hochberg’s approach for controlling the false discovery rate. Genes with an adjusted P-value < 0.05 found by DEGseq were assigned as differentially expressed genes [34]. The unpaired Student’s t-test was used to examine differences in learner and failed-learner group of bees. ANOVA test was used to different the three learning trials of *A*. *mellifera legustica*. For statistical significance real-time PCR data, independent-samples t-test was used. Data are presented as mean ± SD [35, 36].

## Results

### Olfactory learning performances

**Fig 1:**
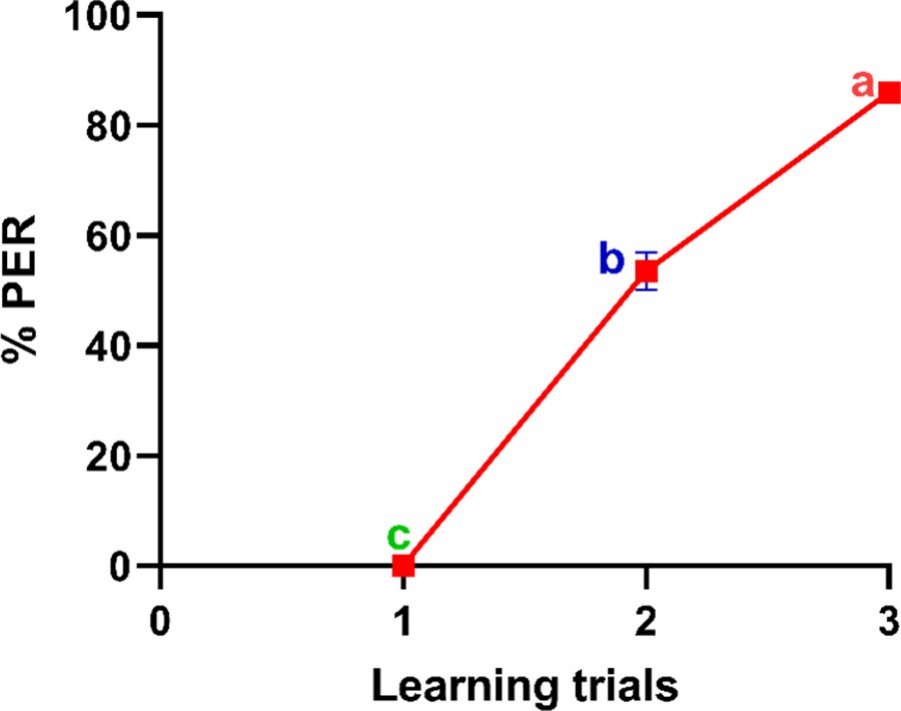
Proboscis extension response percentage of *A*. *mellifera* bees (12-day adult bees) of in odor response and sucrose reward. PER (%) explicit the significantly higher learning response 3^rd^ conditioning trial as compared to 2^nd^ and 1^st^ trials.

Our result revealed that percent responses were lower on the first conditioning trial and increased on subsequent trials, indicating that the bees learned the odor-sucrose association Fig 1. The significant differences among the conditioning trials (T1, T2, T3) were represented in small alphabetical letters (a, b, c). (F 282.184; P < 0.0001), (***p ≤ 0.001, IBM SPSS Statistics 21, ANOVA).

**Fig 2:**
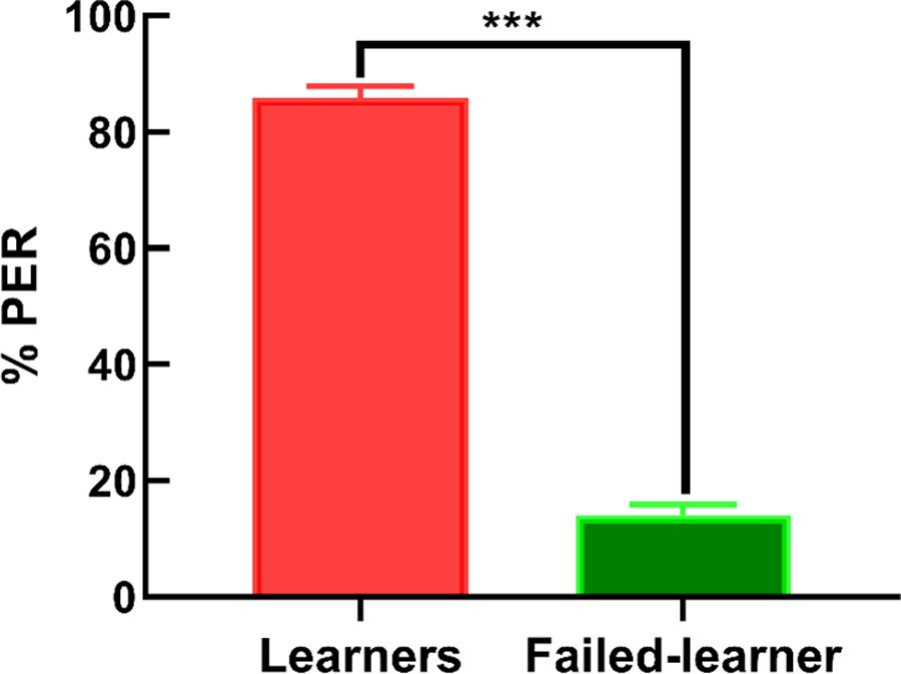
Proboscis extension response learning trials of *A*. *mellifera* bees to odor response and sucrose reward. PER (%) exhibited that learner bees significantly higher learner response as compared to failed learner bees, x-axis and y-axis represents the learner and failed-learner bees and percentage of proboscis extension response respectively.

During the proboscis extension response, we used 251 bees. After conditioning trials, we defined the learner and failed-learner groups. The number of learner bees was “216,” and failed-learner bees were “35” Fig 2. The data of learner and failed-learner group of *A*. *mellifera* bees expressed in (mean ± SD) (t-test, t = 17.531, df = 10, P < 0.0001).

### Sequencing analysis and quality control

Transcriptome analysis of *A*. *mellifera ligustica* bees was performed to understand the transcriptional changes in learner and failed-learner bees. After removing low-quality reads, further than 6.77 billion clean reads with suitable quality were generated in three samples. After cleaning and quality checks, the percentage of Q30 and GC exceeded 92% and 38%, respectively Table 1.

**Table 1:** Summary of data quality control.

Sample name	Raw reads	Clean reads	Q20 (%)	Q30 (%)	GC content (%)
L1	47959604	46643000	97.10	92.31	39.78
L2	41507724	40104884	97.01	92.09	39.47
L3	55067748	53512630	96.96	92.00	38.97
NL1	4960828	47971142	97.07	92.27	39.34
NL2	46229844	45141096	96.81	91.70	38.28
NL3	47197408	46098262	96.97	92.06	38.26

### Analysis of differentially expressed unigenes (DEGs) by RNA-Seq

**Fig 3:**
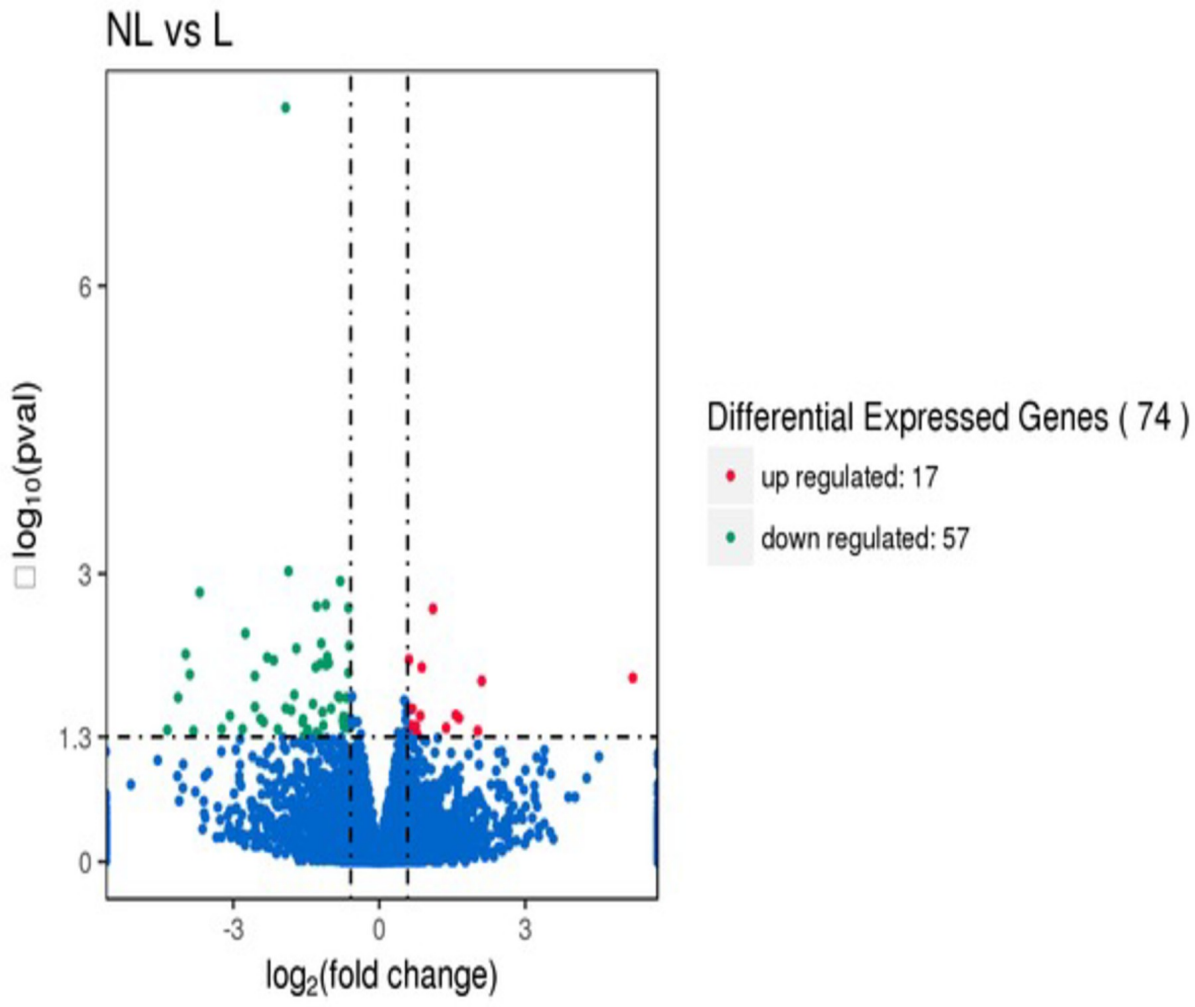
Volcano plot for (DEGs) differentially expressed genes between learner and failed-learner groups.

The average number of clean reads of learner and failed-learner bees were 46.7 and 46.4 million reeds, respectively after, filtering low-quality reads. Approximately 93% of the clean reads were mapped of learner bees and failed-learner bees. In total, 74 genes were identified that were differentially expressed from the learner bees and failed-learner bees (padj < 0.05). Among the 74 differentially expressed genes (DEGs), 57 genes were down-regulated in failed-learner, and 17 genes were upregulated in the brains of learner bees Fig 3.

Up-regulated and down-regulated genes are significantly presented in the red part (red dots) and green part (green dots) respectively (padj < 0.05). There was no differential expression was found presented in blue color between the learner and failed-learner groups (padj > 0.05).

**Fig 4:**
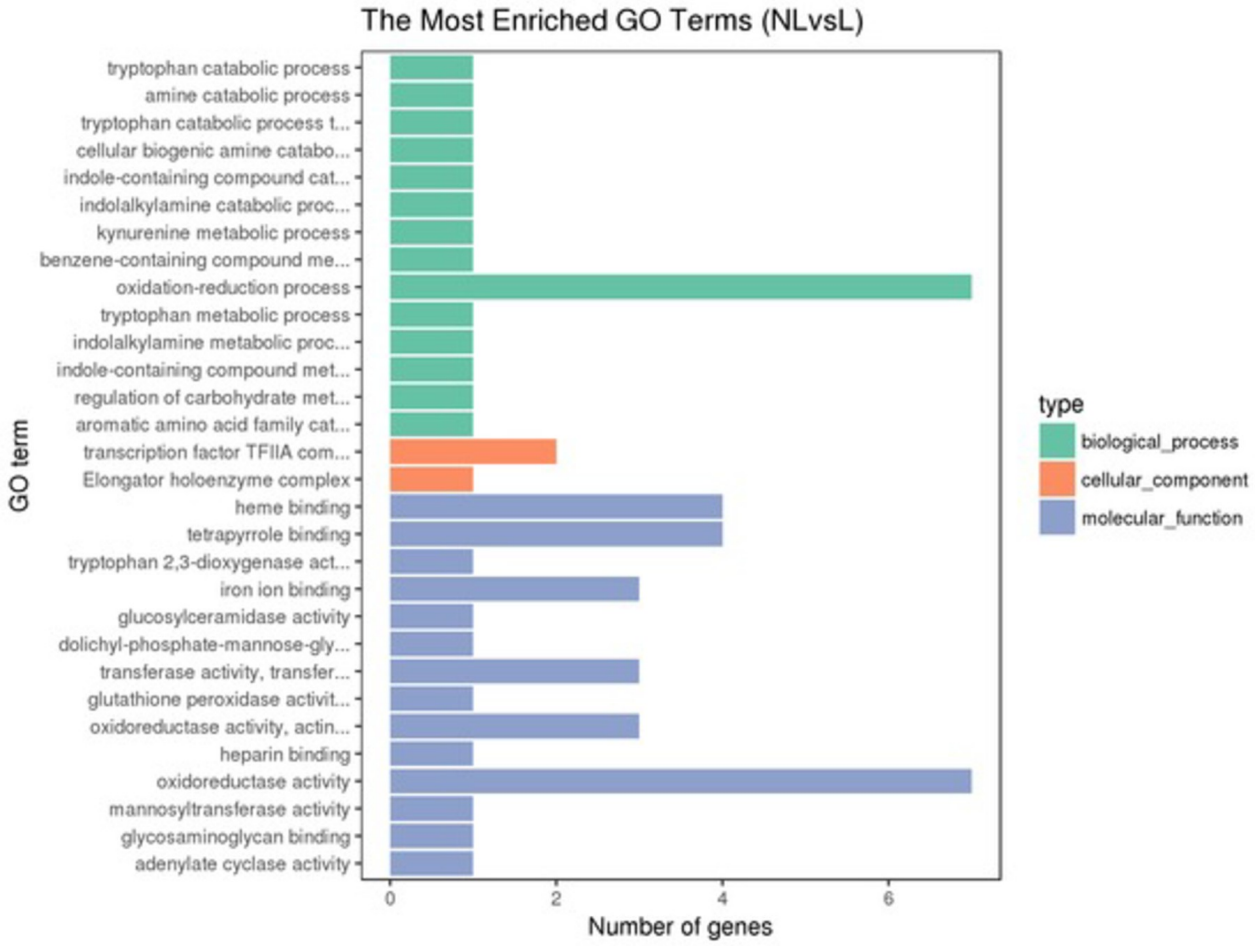
Gene ontology functional classification.

The x-axis is gene ontology (GO) terms enriched, and the y-axis represents the differential expression genes. The (GO) terms are extensively used to describe the biological process, molecular function, cellular component of genes. GO enrichment bar chart is used to clarify the differentially expressed genes enriched GO terms and the counts of genes for each GO term. The most enriched 30 GO terms are shown in Fig 4.

### Validation by qRT-PCR

**Fig 5:**
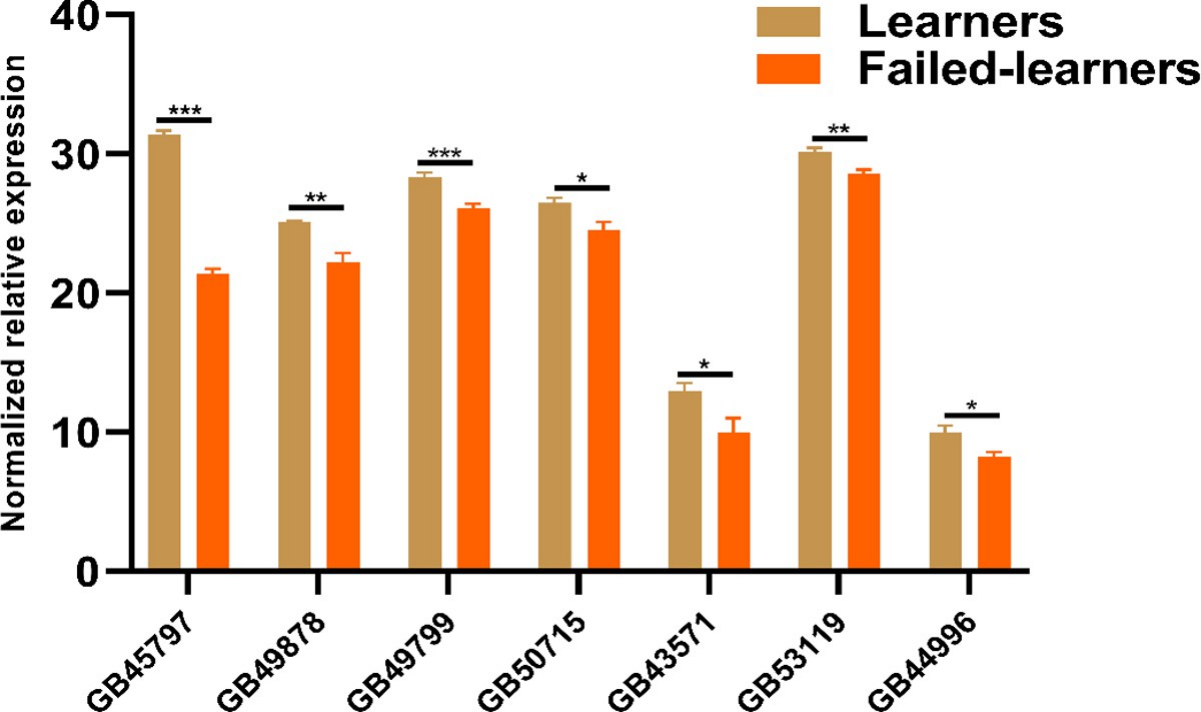
qPCR analysis of differentially expressed genes between learner and failed-learner bees. Independent t-test, * P<0.05, ** P< 0.01.

The relative expression level of seven DEGs is associated with brain development, integumentary system, storage proteins, sensory processing, and neurodegenerative disorder, furthermore, confirmed by qRT-PCR Fig 5. The distinct expression of seven DEGs by qRT-PCR agreed substantially with RNA-Seq genes expression data. The relative gene expression levels of the seven DEGs were significantly lower in failed-learner honeybees than learner bees (P< 0.05; P< 0.01) Fig 5. The expression levels of caste differentiation and transmembrane transport genes MRJP1 (GB45797) and battenin (GB49799) in learner bees were significantly higher than in failed-learner honeybees. The expression levels of immune system genes, rhythmic behaviors, detoxification, storage protein including probable cytochrome P450 6a14 (GB49878), protein unc-80 homolog (GB50715), esterase A2 (GB43571), apidermin 2 (GB53119), hexamerin 110 (GB44996) in learner bees, were significantly high in learner honeybees.

The results confirmation of the seven DEGs showed that the qRT-PCR values were well correlated and confirm the significance of the transcriptome data because selected genes are also involved in several functions such as caste differentiation, transmembrane transport, and immune system in the brains of honeybees.

## Discussion

The study was conducted to identify the gene whose expression differs in the learner and fail-learner bees following proboscis extension response, olfactory learning was performed on restrained individuals using the conditioned proboscis extension response. The results revealed that the learning performance of *A*. *mellifera* (12 days) showed significantly different between learner and fail-learner bees. We obtained approximately 46.7 and 46.4 million clean reads (average) from the brains of learner bees and fail-learner bees, respectively. The results showed that 74 differentially expressed genes were identified between learner and fail-learner bees, including 57 up-regulated and 17 down-regulated genes. RNA sequencing data were confirmed by qRT-PCR. Transcriptome analyses revealed that specific genes in learner and fail-learner bees either down-regulated or up-regulated involving brain development and learning behavior. Our finding suggests that the downregulation of brain genes involved in the integumentary system, storage proteins, brain development, sensory processing, and neurodegenerative disorder may reduce olfactory discrimination and olfactory sensitivity fail-learner bees. Olfactory cognition triggered by odor (smell, scent) experience, the olfactory pathway has been well documented, and adaptive behavior depends on learning to improve experiential choices. A stable combination of sugar reward and odor will reliably provoke a suitable behavioral response (PER). Even "simple" classical conditioning protocol and experiment leads to many brain changes including changes in the experiment pattern [37], variations in basic odor proceeding [38–40] and distinctive traces of associative memory [41]. The protein portion consists primarily of major royal jelly proteins (Mrjps), among them nine (Mrjp1-9) encoding genes identified [42–44]. The (Mrjp 1–9) encoding genes are present in the *Apis* genus and other species, including, *Megachile rotundata*, *Nasonia vitripennis*, and in ants and several bumble bees [43]. Mrjps also found in Polistes canadensis [45]. The first Mrjp exhibited species in the Vespidae family [46]. A family of multi-functional proteins, (Mrjps) in bees are directly and indirectly involved in regulating the behavioral, developmental, and physiological processing [43]. Mrjp1 is the most important and abundant protein in royal jelly. Mrjp1 was already demonstrated and expressed in the brain [47–49]. At the same time, prominent expression of mrjp1 in Kenyon cells, which is crucially performed special task in the formation of olfaction learning abilities of honeybees [49]. Honey bees with reduced learning skills did not showed high-levels of mrjp1 expression [47]. The mrjp1 was significantly expressed in the brains of learner group bees involved in learning performance. This finding provides additional evidence and fits well with our results that Mrjp1’s key role in developing honeybee-learning capabilities. The functional and molecular characteristics of Mrjp1 have remained unclear in the same age bees of *A*. *mellifera ligustica*. Besides the expression of the Mrjps in the hypopharyngeal glands (HGs), several investigations were reported using proteomic examines of royal jelly and major royal jelly protein [47, 49–51]. Furthermore, findings on the transcriptome analysis of *A*. *mellifera* L. shown contigs of Mrjps, mainly Mrjp1, in libraries of the honeybee brain [48, 52, 53]. In addition to the expression of these proteins in the (HGs), in the brain of working bees, Mrjp1 also identified [54, 55]. Mrjp1 is an essential royal jelly (RJ) protein [44]. The Mrjp1’s expression showed in the brain [47, 56], and more particularly in the mushroom bodies (Kenyon cells) of the brain, Kenyon cells involved in the learning and memory formation [49]. Juvenile NCL (JNCL) is a hereditary (an autosomal recessively) neurodegenerative disturbance that is concerned with a mutation in the battenin gene. JNCL has been considered by the accretion of motor decline [57] of neurons and diminished brain mass. CLN3 is a hereditary disease that affects the nervous system. In the human being, with CLN3 disease, faced difficulty learning the ability of new information and started losing previously acquired skills [58–62]. Due to a mutation in CLN3, there are clear indications of cognitive impairment, depressed mood, anxiety, loss of learning abilities, attention, loss of memory, feeding, and adaptive skills [51, 63–66]. The CLN3/battenin gene mutations lead to Juvenile (neuronal ceroid lipofuscinosis) NCL, cognitive decline, and movement disorders [67–69]. Our outcomes provide significant clues about the role of battenin in the brain during learning trials, especially the involvement of learning behaviors. According to different studies, lacking or mutation in the battenin gene causing abnormalities in mice brain, changes in behavior such as learning and memory impairment, and also minimize the involuntary activity level [59]. The CYPs were identified in the brains of several species as well as the honeybee, rat, human, monkey, dog, and mouse [70, 71]. In the central nervous system, CYPs can also play a significant role in regulating brain activity, learning behavior, CNS disease susceptibility, and consequences of treatment [72]. Cytochromes P450 are expressed in the brain, liver, and including other organs [73]. After completion of brain development, it is far from static. The brain evolves to stimuli that underlie learning, behavior, and memory functions throughout life, and the ability of the brain overcome to damage [74]. The CYP450 affects isoforms in the metabolism of neurosteroids, cholesterol, and neurotransmitters, therefore their potential participation in animal behavior, such as in learning and memory, cognitive processes, schizophrenia stress, depression. CYP450 facilitated alternative serotonin and dopamine pathways, synthesis may play a significant role in the local production of such neurotransmitters in brain regions, and integration of local alternate neurotransmitters can be extremely important in the brain [75]. Recent work supports this possibility that cytochrome P450 (GB49878) is essential for brain development and has a significant role in the learning of bees. Cytochrome P450 has a critical function in the hippocampus (a necessary part of the brain of invertebrate and vertebrates) [76]. The hippocampus is mainly associated with mechanisms of learning and memory have a particular contribution as neuromodulators to quick action on memory formation [77, 78]. Several individuals have behavioral issues, such as self-stimulatory and repetitive behaviors and sensitivity. Mutation in UNC80 shows tactile learning and hypersensitivity to stimuli. The presence of UNC80 is crucial for the channel function of NALCN [79–82]. Mutation of the UNC80 in mouse brains demonstrates the interdependency of NALCN sensitivity to extracellular calcium in the channel complex. UNC80 is essential for NALCN sensitivity to extracellular calcium [83]. We reported the first time that UNC80 is indirectly involved in the learning and memory of honeybees and causes a lack in the cognitive task [84]. Animals require intact learning and well-kept memory for the survival of various environmental circumstances. Circadian rhythms are fundamental biological concepts that are conserved in multiple organisms. A network of circadian neurons in the fruit fly Drosophila melanogaster drives daily rest and activity rhythms [85]. In the central nervous system and on the periphery, including in the hippocampus, circadian rhythmicity in gene expression and physiological processes was observed. The hippocampus is a brain-critical region for learning formation. UNC80 is essential in drosophila to promote circadian behavioral rhythm [86]. Our recent findings and previous behavioral facts suggest genes involved in caste differentiation, depression; facilitate pathways for neurotransmitters, integumentary system, storage proteins, brain development, sensory processing, and neurodegenerative disorder, which may cause impairment learning and reduce the learning performance in failed-learner bees [87–90].

To the best found of knowledge, this is the first study to be examined transcriptome expression patterns in the brain of honeybees (12-days old), revealing the odor learning behaviors between learner and failed-learner. The profiles of gene expression underlying cognitive patterns indicate that genes are associated with brain development; integumentary system, storage proteins, sensory processing, and neurodegenerative disorder were down-regulated significantly in failed-learner brains of the bees. These results together show that most differentially expressed genes were significantly down-regulated in failed-learner group. Down-regulated brain genes were involved in multifunction, such as mood regulation, stress, anxiety, integumentary system, storage proteins, brain development, sensory processing, a neurodegenerative disorder, and circadian rhythms. It leads to reduced learning skills.

## Conclusion

In our study, we obtained a total of 74 differentially expressed genes through DEG analysis after olfactory learning trials, among them few were reported to be important genes involved in olfactory learning behavior. As a result, we obtained worthy information. These genes also provide important clues for future studies related to olfactory learning response in bees and greatly expanded the scope of molecular mechanism related to olfactory learning behavior in bees.
